# Inequalities in Untreated Root Caries and Affordability of Dental Services among Older American Adults

**DOI:** 10.3390/ijerph17228523

**Published:** 2020-11-17

**Authors:** Fatma Badr, Wael Sabbah

**Affiliations:** 1 Department of Oral Diagnostic Sciences, Faculty of Dentistry, King Abdulaziz University, P.O. Box 80209, Jeddah 21589, Saudi Arabia; ffbadr@kau.edu.sa; 2 Faculty of Dentistry, Oral & Craniofacial Sciences, Dental Public Health, King’s College London, Denmark Hill Campus, 2nd Floor Dental Extension, Bessemer Road, Denmark Hill, London SE5 9RS, UK

**Keywords:** aged, dental care, socioeconomic factors, risk factors, root caries

## Abstract

The growing geriatric population is facing numerous economic challenges and oral health changes. This study explores the relationship between affordability of dental care and untreated root caries among older American adults, and whether that relationship is independent of ethnicity and socioeconomic factors. Data from 1776 adults (65 years or older) who participated in the National Health and Nutrition Examination Survey (NHANES) were analyzed. The association between affordability of dental care and untreated root caries was assessed using logistic regression models. Findings indicated that untreated root caries occurred in 42.5% of those who could not afford dental care, and 14% of those who could afford dental care. Inability to afford dental care remained a statistically significant predictor of untreated root caries in the fully adjusted regression model (odds ratio 2.79, 95% confidence interval: 1.78, 4.39). Other statistically significant predictors were gender (male), infrequent dental visits, and current smoking. The study concludes that the inability to afford dental care was the strongest predictor of untreated root caries among older Americans. The findings highlight the problems with access to and use of much needed dental services by older adults. Policy reform should facilitate access to oral healthcare by providing an alternative coverage for dental care, or by alleviating the financial barrier imposed on older adults.

## 1. Introduction

In the United States of America (USA), one in seven people are older than 65 years of age. By 2060, the proportion of older American adults, aged 65 and over, is projected to almost double, reaching 98 million [1]. The unmet demand for dental services is likely to worsen with this pattern of population growth [2]. Increasing age is associated with health issues, including oral health changes. These changes include increased decayed, missing, or filled teeth (DMFT) [3], root caries [4], xerostomia [5], and limited access to oral health care [6]. Edentulism has significantly declined over the past decades, especially in economically developed countries, leading to older individuals retaining more of their teeth for longer; however, the trend of root caries remains ambiguous [5,7].

Oral health inequalities are usually defined as avoidable and unfair differences in oral health between different groups of people [8]. In the USA, oral health inequalities exist in coronal caries, tooth loss, and edentulism [5,9]. These oral health outcomes tend to be worse among poorer, less educated, and ethnic minorities, even after controlling for individual behavioral factors [10]. Earlier studies have repeatedly reported the persistence of socioeconomic inequalities in oral health among older American adults [11], and highlighted the role played by different social and economic indicators in poor oral health [12].

Older individuals that have retired often face financial barriers that hinder access to dental services [6]. Unsurprisingly, the cost barrier for dental care relatively declined since 2010 for younger individuals but remained high among older adults [13]. The affordability of a specific healthcare service, such as oral health, is determined by the ability to pay and the willingness to pay [14]. This simply means that among older adults, two factors are at play; on one hand the decline of income and loss of private insurance after retirement affects an individual’s ability to pay for dental services, and on the other hand, the increasing need for other health and/or social services likely places dental care at the bottom of an individual’s priorities, hence affecting willingness to pay for dental care. The inability to access and inability to afford dental services exacerbates the problem of root caries among older adults [7]. Furthermore, an earlier study found that making dental care affordable would have an impact on disability-adjusted life years (DALYs) [15]. The aim of this study is to assess whether the inability to afford dental care is associated with the prevalence of untreated root caries among older Americans, and whether this relationship is independent of ethnicity and socioeconomic factors.

## 2. Methodology

### 2.1. Data Source

Data from the National Health and Nutrition Examination Survey (NHANES) 2015/2018 was used. The NHANES uses complex, stratified, multistage probability sampling to obtain a representative sample of the noninstitutionalized civilian resident population of the USA. The data used was a compilation of the most recent surveys released in 2015–2016 and 2017–2018. All participants provided written informed consent. The aforementioned two-year periods were combined in order to improve the power and reliability of estimates. Each participant of this comprehensive survey underwent a home-administered interview, as well as a clinical examination in a mobile examination center (MEC). Clinical assessment was performed by trained and calibrated dental examiners. More information about the NHANES can be found on the website http://www.cdc.gov/nchs/nhanes.htm. The data are available on the CDC website and can be freely downloaded [16]. Ethical approval for the NHANES waves 2015–2016 and 2017–2018 was obtained from the NCHS Research Ethics Review Board [16]. For this secondary data analysis of NHANES data, no further ethical approval is needed.

### 2.2. Population and Sample

In this study, data analysis was limited to adults aged 65 and older who participated in either the NHANES 2015–2016 or 2017–2018 and completed the root caries assessment component. The total number of American adults included in the analysis was 1776.

### 2.3. Variables

The main outcome variable was untreated root caries, a dichotomous variable indicating whether the participant has untreated root caries or not. Root caries assessment was carried out following a strict protocol by trained and calibrated dentists. A mirror, a No. 23 explorer, and compressed air were used for the examination. Root caries was defined as discoloration and softness apical to the cementoenamel junction (CEJ), based on visual and tactile evidence. Inability to afford dental care was the main explanatory variable. Participants were asked whether affordability of dental services was the main reason for not using dental services when needed. Age was used as a continuous variable. Gender was used as a binary variable (male or female). Ethnicity/race was grouped following NHANES classification into: Mexican American, Non-Hispanic White, Non-Hispanic Black, Other Hispanic, and others. Marital status was used as a dichotomous variable, as individuals were grouped into either married/live with a partner or single/widowed/divorced/separated.

Poverty to income ratio (PIR) was calculated by dividing the family income by the poverty level, specific to each state and depending on family size. This ratio is more reliable than income as it adjusts for changing monetary value over time. In this study, PIR was used as a continuous variable for a more accurate assessment of poverty. Health insurance included any governmental or private health insurance such as Medicare, and was used as a dichotomous variable (present or absent). A variable indicating inability to seek treatment because dental insurance did not cover the service was also included in the analysis. Participants were assigned into one of three groups based on self-reported educational attainment: less than high school, high school diploma or equivalent, or university degree or more.

Flossing was treated as an ordinal variable based on the number of days an individual used dental floss per week (0–7). Number of remaining teeth was used as an ordinal variable (0–28), based on clinical examination. Individuals were assigned into a group based on their last dental visit: either within the last two years/more often, or less often/never. The sample was divided into 3 groups based on smoking status (never, former, or current). Diabetes was reported based on participants’ response to the NHANES questionnaire about physician-diagnosed diabetes (yes or no).

### 2.4. Data Analysis

Statistical analysis was done using STATA^®^ (StataCorp. 2019. Stata Statistical Software: Release 16. College Station, TX, USA: StataCorp LLC) to reveal descriptive and inferential statistics using survey command and accounting for strata, primary sampling units, and mobile examination weight. The distribution of all variables included in the analysis was assessed. Percentages of untreated root caries within each group was examined using a chi-square test. Two sets of logistic regression were performed using affordability of care to predict root caries, while controlling for confounders and covariates.

## 3. Results

The distribution of demographic, socioeconomic, and behavioral characteristics within the sample is summarized in Table 1. The mean age of included participants was 72.4, and the range was 65 to 80 (individuals older than 80 were recorded as 80). In the semi-adjusted model, after adjusting for gender, age, and ethnicity, self-reported inability to afford care had an odds ratio (OR) of 4.68 (95% confidence interval (95% CI) 3.33, 6.57) for having untreated root caries. Non-Hispanic Blacks had higher odds for untreated root caries (OR 1.88, 95% CI 1.17, 3.01). However, this was attenuated in the fully adjusted model predicting untreated root caries. On average, females had lower odds for untreated root caries (OR 0.60, 95% CI 0.48, 0.77) and (OR 0.67, 95% CI 0.48, 0.94) in the semi-adjusted and fully adjusted models, respectively.

In the fully adjusted model, inability to afford care continued to be statistically significant in predicting untreated root caries (OR 2.79, 95% CI 1.78, 4.39); however, age and ethnicity were rendered statistically insignificant. Individuals with more teeth had lower odds of having root caries (OR 0.94, 95% CI 0.92, 0.096). Individuals who visited a dentist within the last two years were less likely to have untreated root caries (OR 0.64, 95% CI 0.42, 0.97). Current smokers had higher odds of having untreated root caries (OR 2.04, 95% CI 1.04, 4.01) (Table 2).

## 4. Discussion

This study examined the association between the affordability of dental care and untreated root caries among older American adults. Inability to afford dental care was the strongest variable predicting untreated root caries in this analysis, even after accounting for a number of confounders. The results support the abundant literature reporting the detrimental impact of low socioeconomic status on oral health [2,17,18,19,20].

This study revealed that root caries affected 16% of older American adults. Griffin et al. estimated an annual root caries incidence of 25.8% in 2004 [21], and Rozier et al. reported a much greater root caries prevalence of 36% [22] in 2017; however, both reports were based on much older data. This is in line with the general trend of declining caries among children and younger adults, and declining tooth-loss trends among older American adults. Our findings demystify the reported lack of an obvious trend in root caries that was brought to light by Dye et al. [5].

The majority of older adults lose their private insurance after retirement, and they also have to cope with a drop in their income. While many older adults rely on Medicare, which does not cover dental care, some might purchase additional insurance plans that cover the bare minimum dental care, if any. Simultaneously, their dental needs continue to increase as they retain their teeth into old age and develop more dental problems including periodontal diseases and coronal and root caries, which further aggravate the situation. The strong association between affordability of dental care and untreated root caries observed in this analysis undoubtedly highlights the problem of increased dental needs and the decrease in/lack of dental insurance coverage faced by older American adults.

We used several indicators to determine socioeconomic position (SEP), such as poverty to income ratio (PIR), health insurance, dental insurance coverage, education level, and affordability of care. Affordability of dental care appeared to be a stronger and more precise economic indicator of untreated root caries than income and insurance coverage. Unsurprisingly, the mean PIR in this sample was significantly lower in the root caries group. However, this association was attenuated after adjusting for other factors. This finding is consistent with other studies which found that income was not a statistically significant predictor of root caries among older individuals [23]. Although we accounted for general health insurance and whether dental insurance covered for a specific procedure, inability to afford dental care was the only statistically significant predictor of untreated root caries in the fully adjusted regression model. Numerous adults are still unable to afford dental care despite having health insurance, highlighting the problem of excluding dental care from different insurance schemes for older adults, including Medicare.

The association between educational attainment and root caries was not statistically significant in the fully adjusted model. Similarly, within American adults the impact of educational level on root caries, although significant for middle-aged adults, was insignificant for those 65 years and older [24]. A possible explanation may be that the influence of educational attainment on oral health fades in this age group that is composed mainly of retirees, or that other factors become more prominent, such as affordability of dental care.

This study found that increasing age is associated with higher odds of having root caries. Other studies found a similar impact of age on the prevalence and severity of root caries [23]. Leake et al. found that an annual addition of 0.19 on the decayed and filled root surfaces index (RDFS) is to be expected for each additional year in dentate individuals [25]. Declining cognitive abilities and lack of motivation may explain the increased risk for the elderly [26]. In addition, the increased prevalence of gingival recession as people age [4] supports our findings, since root caries require exposed roots in order to take place.

Several studies found that ethnic minorities or African Americans were more prone to develop caries than Whites [9,23,27]. Kim et al. found that racial inequalities increase with age in oral health based on the NHANES 1999–2004 [27]. Conversely, this study demonstrated that racial inequalities were attenuated after controlling for factors such as affordability and were rendered statistically insignificant. This implies that race or ethnicity, per se, may not be a risk factor for root caries, but rather a confounding factor.

Gender predilection for root caries is consistent in the literature. Females had lower odds of having untreated root caries in our study. This is consistent with a systematic review which reported that males were unequivocally more prone to develop root caries than females [7].

Losing a spouse or partner at an old age has a psychological impact that may cripple oral hygiene practice or encourage a person to acquire an unhealthy habit such as smoking. Tsakos et al. found that, based on NHANES 1999–2004 data on older adults, widowed or divorced individuals had fewer sound or filled teeth than married or coupled individuals [12]. Our study found that marital status was not associated with root caries in either the crude or the adjusted models. Similarly, based on Chinese and European subjects, Persson et al. did not find a significant impact of marital status on periodontal health [28]. Our results suggest that marital status does not currently have the same impact it used to. However, we did not analyze data on other social relationships, such as number of close friends, which might still have an impact on the prevalence of untreated root caries.

In this sample, current smokers had twice the odds of having root caries when compared to non-smokers, after adjusting for confounding factors. This supports many studies that report smoking as the strongest modifiable risk factor in developing periodontal disease and root caries [7,29].

Flossing frequency was not a statistically significant factor in predicting root caries. However, those that had visited the dentist within the last two years had lower odds of having untreated root caries than those who never visited the dentist. A higher number of teeth was associated with lower odds of having root caries. A possible explanation may be that those who retain more teeth take better care of them, and are therefore less prone to root caries. Gilbert et al. found that the pattern of dental visits and number of teeth were able to predict coronal caries [30]. Similarly, they found that attitude towards flossing (but not frequency of flossing) also predicted caries incidence. The results also showed that the occurrence of root caries was higher among diabetic patients. This is scientifically plausible since diabetic patients suffer from poor periodontal health and loss of attachment, with consequential root exposure [31] making them vulnerable to the development of root caries.

To the best of our knowledge, this is the first study to examine the relationship between affordability of dental care and root caries among senior American adults. Nevertheless, the study has several limitations worth mentioning. The cross-sectional design of the survey does not allow conclusion on causality or temporality. The small percentage of participants who reported an inability to afford dental care is another limitation that could have affected the precision of the estimates. Despite accounting for dental visits, there is no information about the treatment received during the visit. Although ethnically diverse and nationally representative, the sample excludes elderly persons living in long-term care facilities, whom often demonstrate worse oral health [32]. Contextual factors such as living in rural or urban areas were not included in the analysis; however, other indicators were used, such as poverty to income ratio and educational level, which are common indicators for socioeconomic status in health research conducted in America [33]. Data on previous caries experience and on oral hygiene behaviors, aside from flossing, were not available in the survey. While we acknowledge oral hygiene and diet as very important risk factors for caries and root caries, the fact that the current analysis used untreated root caries as the main outcome highlights the use of dental services and their affordability as a crucial determinant of existing untreated root caries.

An important observation in the current analysis is the persistence of a strong and significant association between the affordability of dental care and untreated root caries, after accounting for other socioeconomic factors and ethnicity. This indicates the importance of the availability of health insurance premiums that cover dental care among older people to facilitate equitable oral health.

## 5. Conclusions

Inability to afford dental care when needed is significantly associated with untreated root caries, after adjusting for confounding factors, within older American adults. Unlike ethnicity/race, inability to afford care remained a statistically significant predictor for untreated root caries in the fully adjusted regression model. Other statistically significant predictors were gender (male), fewer teeth, fewer dental visits, and being a current smoker. Policy reform should facilitate oral healthcare by alleviating the financial barrier imposed on this vulnerable group.

## Figures and Tables

**Table 1:** Distribution of all variables included in the analysis and percentage of untreated root caries, in older American adults aged 65 +, National Health and Nutrition Examination Survey (NHANES) 2015–2018, (*n* = 1776).

Characteristic		Sample (% or Mean) (5% CI)	Percentage with Untreated Root Caries, or Mean within Those with Untreated Root Caries (95% CI)	*p*-Value *
Age (mean)		72.4 (71.9, 72.9)	73.4 (72.5, 74.3)	0.034
Gender	Female	55.1 (53.0, 58.0)	13.4 (11.0, 16.0)	0.002
	Male	44.9 (42.0, 47.0)	19.03 (16.0, 23.0)	
Ethnicity	Mexican American	4.0 (2.0, 6.0)	24.0 (14.0, 39.0)	0.002
	Non-Hispanic White	4.0 (2.0, 5.0)	17.0 (13.0, 22.0)	
	Non-Hispanic Black	80.0 (75.0, 84.0)	14.0 (12.0, 17.0)	
	Other Hispanic	6.0 (5.0, 9.0)	26.0 (19.0, 34.0)	
	Others	6.0 (5.0, 9.0)	22.0 (16.0, 29.0)	
Marital status	Married	63.3 (58.5, 67.9)	13.9 (10.6, 18.2)	0.080
	Single	36.7 (32.1, 41.5)	19.4 (15.7, 23.6)	
Affordability	Able to afford (AA)	93.2 (91.4, 94.7)	14.0 (11.9, 16.4)	0.000
	Unable to afford (UA)	6.8 (5.3, 8.6)	42.5 (34.4, 50.9)	
Poverty to income ratio (mean)		3.3 (3.1, 3.5)	2.7 (2.4, 3.0)	0.000
Health insurance	Insured	98.6 (97.8, 99.2)	15.7 (13.3, 18.6)	0.090
	Non-insured	1.4 (0.8, 2.2)	29.7 (14.6, 51.1)	
Insurance did not cover procedure	No	97.6 (96.8, 98.1) 2.4 (1.8, 3.2)	15.1 (12.7, 17.8) 49.5 (37.3, 61.7)	0.000
	Yes			
Educational level	Less than high school	10.9 (8.6, 13.8)	26.4 (20.8, 32.9)	0.000
	High school	53.0 (47.9, 58.1)	17.4 (14.4, 20.9)	
	University level	36.1 (30.4, 42.1)	10.6 (7.6, 14.6)	
Flossing (mean number of days per week)		4.1 (3.9, 4.3)	3.1 (2.6, 3.6)	0.000
Number of teeth (mean)		21.6 (20.8, 22.3)	16.7 (15.4, 18.0)	0.000
Dental visits	Never or >2 years ago	18.8 (15.7, 22.5)	30.0 (24.1, 36.6)	0.000
	More often	81.2 (77.5, 84.3)	12.7 (10.5, 15.2)	
Smoking	Never smokers	52.2 (48.8, 55.6)	13.3 (10.2, 17.1)	0.001
	Former smokers	41.3 (38.0, 44.6)	16.3 (12.4, 21.1)	
	Current smokers	6.5 (5.2, 8.3)	35.3 (23.7, 48.8)	
Diabetes	No	77.1 (74.0, 79.9)	14.0 (11.5, 17.0)	0.002
	Yes	22.9 (20.1, 26.0)	22.4 (17.8, 27.7)	

* *p*-Value for chi-square and *t*-test.

**Table 2:** Logistic regression odds ratios for factors associated with untreated root caries in older Americans aged 65 +, NHANES 2015–2018, (*n* = 1776).

Root Caries (RC)	Model 1	Model 2
	(Semi Adjusted)	(Fully Adjusted)
	OR (95% CI)	OR (95% CI)
Gender (ref: male)	0.60 *** (0.48, 0.77)	0.67 * (0.48, 0.94)
Age	1.05 ** (1.01, 1.10)	1.03 (0.99, 1.07)
Ethnicity (ref: White)		
Mexican American	1.64 (0.78, 3.42)	1.12 (0.53, 2.36)
Other Hispanic	1.08 (0.70, 1.66)	0.69 (0.46, 1.03)
Non-Hispanic Black	1.88 ** (1.17, 3.01)	1.00 (0.62, 1.62)
Others	1.51 (0.93, 2.46)	1.13 (0.59, 2.17)
Could not afford the cost	4.68 *** (3.33, 6.57)	2.79 *** (1.78, 4.39)
Marital status (ref: married)		1.17 (0.65, 2.11)
Dental insurance did not cover the procedure		2.05 (0.94, 4.47)
Not covered by any health insurance		1.47 (0.40, 5.38)
Educational level (ref: < high school)		
High school diploma, or some college		0.96 (0.65, 1.43)
University degree or more		0.98 (0.55, 1.75)
Poverty to income ratio		0.97 (0.83, 1.15)
Flossing		0.96 (0.90, 1.01)
Number of teeth		0.94 *** (0.92, 0.96)
Last dental visit within 2 years (ref: never/ > 2 years ago)		0.64 * (0.42, 0.97)
Smoking (ref: never smoked)		
Former smokers		0.99 (0.62, 1.60)
Current smokers		2.04 * (1.04, 4.01)
Diabetes (ref: no)		1.35 (0.92, 1.97)

*p*-Values: * *p* ≤ 0.05, ** *p* ≤ 0.01, *** *p* ≤0.001. Model 1 adjusted for age, gender, and ethnicity. Model 2 additionally adjusted for marital status, dental insurance, health insurance, education level, poverty to income ratio, flossing, number of teeth, last dental visit, smoking, and diabetes.
